# Unmanned Aerial Vehicle Object Tracking by Correlation Filter with Adaptive Appearance Model

**DOI:** 10.3390/s18092751

**Published:** 2018-08-21

**Authors:** Xizhe Xue, Ying Li, Qiang Shen

**Affiliations:** 1 School of Computer Science and Engineering, Northwestern Polytechnical University, Xi’an 710129, Shaanxi, China; xuexizhe@mail.nwpu.edu.cn; 2 Institute of Mathematics, Physics and Computer Science, Aberystwyth University, Aberystwyth SY23 3DB, UK; qqs@aber.ac.uk

**Keywords:** UAV video, visual tracking, correlation filter, cellular automata, adaptive appearance model

## Abstract

With the increasing availability of low-cost, commercially available unmanned aerial vehicles (UAVs), visual tracking using UAVs has become more and more important due to its many new applications, including automatic navigation, obstacle avoidance, traffic monitoring, search and rescue, etc. However, real-world aerial tracking poses many challenges due to platform motion and image instability, such as aspect ratio change, viewpoint change, fast motion, scale variation and so on. In this paper, an efficient object tracking method for UAV videos is proposed to tackle these challenges. We construct the fused features to capture the gradient information and color characteristics simultaneously. Furthermore, cellular automata is introduced to update the appearance template of target accurately and sparsely. In particular, a high confidence model updating strategy is developed according to the stability function. Systematic comparative evaluations performed on the popular UAV123 dataset show the efficiency of the proposed approach.

## 1. Introduction

Recent years have witnessed significant development in the field of computer vision. An enormous amount of research effort has gone into vision-based tasks, such as object tracking [1,2,3], recognition [4,5] and saliency detection [6]. As an important field of computer vision, visual tracking [7,8,9,10,11] plays an active role in a wide range of applications, in which tracking using UAVs is a very promising one. Since the camera can follow the target based on visual feedback and meanwhile change its orientation and position to improve the tracking performance, aerial tracking [12] is widely applied to a diverse set of objects, which cannot be physically or persistently tracked from the ground, such as humans, animals, cars, boats, etc. Apart from those related to surveillance, a large number of new applications based on aerial tracking have been applied including infrastructure inspection [13], person following [14] and aircraft avoidance [15]. However, compared with static tracking systems, aerial tracking requires the ability of analyzing a dynamic scene and handling new challenges posed on the UAV videos.

UAV tracking usually requires long-term tracking, since the camera can follow the target in contrast to the static surveillance scenario. To alleviate the model drifting and handle occlusion problem, the TLD tracker [1] combined the traditional tracking algorithm with the detection algorithm to make the system more reliable through an online learning mechanism. The tracking algorithm was based on the optical flow method, and the detection algorithm generated numerous candidate regions where each region must be accepted by three filters to become the detection result. The detection algorithm re-initialized the tracker when the tracking algorithm failed. However, it is difficult for TLD to meet the requirement of a large number of frames per second without resorting to parallel computation on a standard PC in dealing with real-time processing tasks. From this viewpoint, correlation filters [16,17,18,19,20,21,22] show their strengths both in speed and accuracy, where tracking problem is converted from time domain to frequency domain with fast Fourier transform (FFT). In so doing, convolution can be substituted with multiplication in an effort to achieve fast learning and target detection. Similar to TLD, the redetection procedure was carried out based on an online random fern classifier in the long-term correlation tracking (LCT) method [16], and the training samples were collected by a k-nearest neighbor (KNN) classifier. Zhu et al. applied the CUR theory to collaborative correlation tracking (CCT) method [17]. The CUR matrix approximation not only computed the low rank property of object representation, but also projected the representation matrix of historical objects to achieve a robust object representation.

By recognizing the success of deep convolutional neural networks (CNNs) on a wide range of visual-recognition tasks, tracking methods based on CNNs have also been developed [23,24,25,26]. Empirical studies using large object-tracking benchmark datasets have shown that such CNN-based trackers perform favorably against methods based on the use of handcrafted features. Notwithstanding, the underlying approach is computationally expensive and often cannot meet the speed requirements for real-time applications. In [24], Held et al. proposed Generic Object Tracking Using Regression Networks (GOTURN) [24] for offline training of a simple feed-forward network that can track generic objects at more than 100 fps with GPU. Yet, if only a CPU is available, the tracker runs at 2.7 fps [24]. A different piece of work showing favorable results for real-time visual tracking is the biologically inspired tracker (BIT) [27], which can extract low-level biologically inspired features and imitate an advanced learning mechanism. Note that BIT operates in real-time since fast Gabor approximation and fast Fourier transform are used for online learning and detection. However, all these methods cannot cope well with challenges presented by UAV videos, which typically involve low resolution, scale variation, aspect ratio change and occlusion. To address the issues we propose a robust tracking approach, which offers two unique advantages:

(1) In order to handle the scale variation and aspect ratio change, a novel propagation method based on cellular automata (CA) is introduced to update the appearance template of target adaptively and sparsely, which benefits from the consistency among similar image pixels.
(2) A new adaptive template update scheme is proposed to further alleviate the problem of model drift which is caused by occlusion or distracter. The effectiveness of this approach is demonstrated through extensive comparisons against other methods.

The rest of this paper is organized as follows: Section 2 discusses relevant previous work on correlation filter, CA and feature selection in correlation tracking. Under the general framework of correlation filter, Section 3 describes our approach. Section 4 presents an evaluation of the proposed approach and a comparative study with state-of-the-art techniques. Section 5 discusses the tracking speed of different methods and assesses the actual effect of CA in the proposed method. Finally, Section 6 concludes this work and points out interesting further research.

## 2. Related Work

### 2.1. Correlation Filter Trackers

Because of their impressive high-speed, correlation filters have attracted a great deal of interests in object tracking. For instance, Bolme et al. [18] have proposed the minimum output sum of squared errors (MOSSE) filter, which works by trying to find the maximum cross correlation response between the model and candidate patch. Henriques et al. [19] exploited the circulate structure and Fourier transformation in a kernel space (CSK), offering excellent performance on a range of computer vision problems. A vector correlation filter (VCF) was proposed by Boddeti et al. [20] to minimize localization errors while improving the tracking speed. Danelljan et al. [28] exploited the color attributes of an object and introduced the color name (CN) features into CSK to perform object tracking. Combining techniques of kernel trick and cycle shift [19], kernelized correlation filter (KCF) [29] entails more adaptive performance for diverse scenarios using histogram of oriented gradient (HOG) features. The DSST tracker [30] learns adaptive multi-scale correlation filters by the use of multi-channel HOG features to handle the scale change of target objects. To learn a model that is inherently robust to both color changes and deformations, Staple [31] combines two image patch representations that are sensitive to challenging factors. SRDCF [32] utilizes a spatial regularization component in the learning process to penalize correlation filter coefficients as a function of their spatial location. Recently, to drastically reduce the number of parameter in the model, Danelljan et al. [33] proposed a factorized convolution operator. Meanwhile a compact generative model of the training sample distribution significantly reduces the memory and time complexity, while providing better diversity of samples.

Whilst many methods exist, as outlined above, these methods do not address the critical issue of aerial tracking. When scale variation and aspect ratio change exist, the traditional correlation trackers only enlarge or narrow the bounding box in equal proportion, which will introduce a great number of background information and finally result in model drifting. In addition, dense updating scheme will also reduce the aerial tracking efficiency when serious occlusion exists.

### 2.2. CA

CA is a dynamic system with simple structure but is of complex self-organizing behavior, as proposed in [34]. Consisting of a lattice of cells with discrete states, the model evolves in discrete time steps according to the definite rules. The current state of the cell and the states of its nearest neighbors make joint efforts to its next state. CA has been applied to simulate the process of various complicated dynamic systems [35].

Specifically, a CA always operates on a lattice of sites $p\in P\subseteq Z^{n}$. A CA is a triplet A=(S,N,δ), where *S* is a non-empty state set, *N* is the neighborhood system, and $\delta :S^{N}\to S$ is the local transition rule. According to the states of the neighborhood cells at previous time step t, this function defines the rule of calculating the state of cell at t+1 time step.

**Figure 1:**
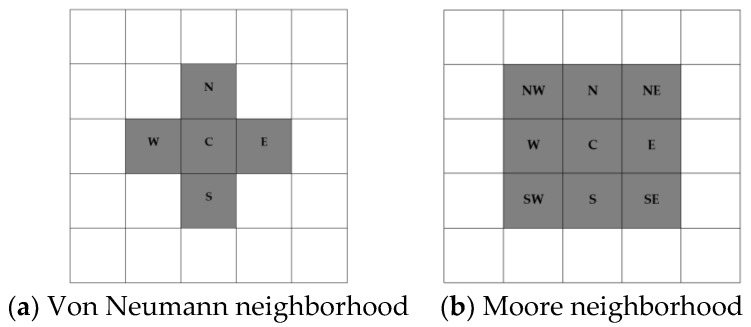
Neighborhood structures.

Von Neumann (Equation (1)) and Moore (Equation (2)) offer two commonly used neighborhood systems. The neighborhood structures associated with these systems are shown in Figure 1:

(1) $$N(p)=\left\{q\in Z^{n}:{\|p-q\|}_{1}:=\sum_{i=1}^{n}|p_{i}-q_{i}|=1\right\}$$

(2) $$N(p)=\left\{q\in Z^{n}:{\|p-q\|}_{\infty }:=\underset{i=\bar{1,n}}{\max}|p_{i}-q_{i}|=1\right\}$$

Given a certain cell, the neighboring cells above and below, and those on the right and left together with the cell itself is called Von Neumann neighborhood of this cell. The radius of the neighborhood definition is 1, as only the next layer is considered.

In addition to the four cells of Von Neumann neighborhood, Moore neighborhood also includes the four next nearest cells along the diagonal. In this case, the radius is equal to 1 also. The cell state $S_{p}$ in our case is actually a triplet $\left(l_{p},F_{p},{\vec{C}}_{p}\right)$, where the label $l_{p}$ denotes the current cell, $F_{p}$ means the ‘strength’ of current cell and ${\vec{C}}_{p}$ is cell feature vector.

### 2.3. Feature Selection in Correlation Tracking

Features play an important role in computer vision, in which gradient and color features are the most widely exploited. In particular, HOG features are the most commonly employed to catch texture and gradient information in object tracking [30,31,32,33], while color measurements can vary significantly over an image sequence due to variations in illuminant, shadows, shading, camera and object geometry. Henriques et al. [19] utilized color attributes or features to obtain excellent results for visual tracking problems. Recent work [36] has verified that there exists a strong complementarity between gradient and color features. On this basis, Danelljan et al. [28] introduced CN features and HOG features together to construct a correlation filter, in an attempt to capture color characteristics and abundant gradient information, considerably improving the tracking performance.

## 3. Proposed Methods

**Figure 2:**
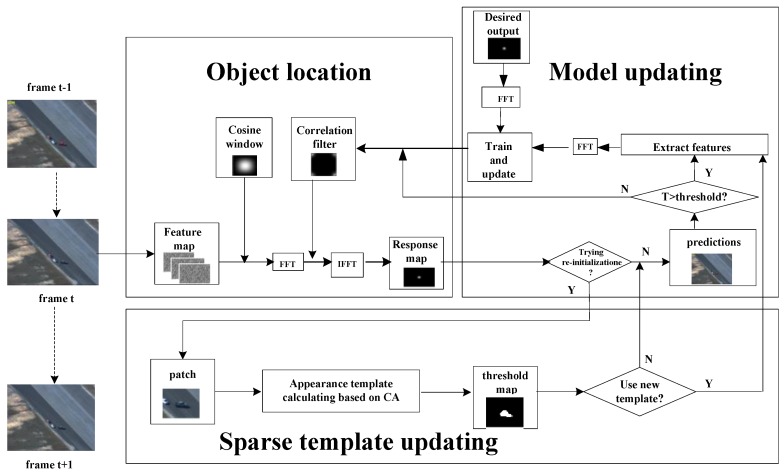
Flowchart of the proposed tracking algorithm.

We aim to develop a robust tracking algorithm that is adaptive to significant appearance change without being prone to drifting. For this, the fused features to be extracted are represented as a multi-dimensional vector of input features (which themselves are each encoded as a one-dimensional vector of multiple real values). Further, CA is introduced to sparsely update the aspect ratio of the bounding box, which makes our method less susceptible to the noise from background. In particular, the adaptive model updating strategy is also put forward in order to achieve better performance. The proposed tracking framework is illustrated in Figure 2. Our algorithm can be divided into three modules: object location, model updating and sparse template updating. In object location, fused features are extracted first, followed by deriving the response map that is calculated by the proposed correlation filter. Target location is estimated by searching for the location of the maximum value within the response map. Afterward, if the conditions are satisfied, CA will be introduced to obtain a new appearance template for re-initialization (while discarding the previous template and training a correlation filter with the current one). In addition, model updating also plays an important role in our method, with the filter being updated only when the indicator *T* is bigger than a given threshold.

### 3.1. Correlation Tracking through Fused Features

The UAV videos are affected by platform motion and jitter, so the color and shape of the target are rapidly changing. Compared with generic object tracking, tracking challenges are amplified in aerial scenarios including abrupt camera motion, distance-induced low resolution, significant changes in scale and aspect ratio, fast moving objects, as well as, partial and full occlusion. Having taken notice of these issues caused by such conditions and related implications on extraction of object details using a single feature, a method fusing CN and HOG features is employed in this work to achieve robust performance in aerial tracking. Furthermore, the aforementioned features are concatenated directly to form a vector as a fused feature descriptor. In this paper, we utilize fused feature vector representation which better fits with the correlation tracking framework. Denote $x_{d}$ as the fused feature vector of a cardinality $d\in R^{D}$. We consider $y_{d}$ as the desired correlation output corresponding to a given sample $x_{d}$. A correlation filter *w* with the same dimensionality of $x_{d}$ is then learned by solving the following minimization problem:

(3) $$w*=\mathrm{argmin}\sum {\left\|w\cdot x_{d}-y_{d}\right\|}^{2}+\lambda {\left\|w\right\|}_{2}^{2}$$

where  λ is a regularization parameter. Note that the minimization problem in Equation (3) is akin to training the vector correlation filters in [20], and can be solved within each individual feature channel using FFT. Let the capital letters be the corresponding Fourier transformed signals. The learned filter in the frequency domain on the d−th (d∈{1,…,D}) channel can be written as:

(4) $$W_{d}=\frac{\bar{Y}⊙X^{d}}{\sum_{i=1}^{D}{\bar{X}}^{i}⊙X^{i}+\lambda }$$

where Y,X,W denote the discrete Fourier transforms (DFT) of y,x,w, respectively; $\bar{Y}$ represents the complex conjugation of Y; and $\bar{Y}⊙X^{d}$ is a point-wise product. Given an image patch in the next frame, the fused feature vector is denoted by $Z\in R^{D}$. The correlation response map is computed by:

(5) $$r=F^{-1}\left(\sum_{d=1}^{D}W^{d}⊙{\bar{Z}}^{d}\right)$$

where the operator $F^{-1}$ denotes the inverse FFT. Then the target location can be estimated by searching for the position of the maximum value of the correlation response map *r*:

(6) $$\left(x\prime ,y\prime )=\underset{a,b}{\arg\max}(R(a,b)\right)$$

### 3.2. Adaptive Appearance Template Updating Based on CA

Typically, the appearance of an object can be divided into shape and scale. The conventional algorithms usually only update the scales, which result in some disadvantages. For example, if the aspect ratio of template cannot adjust to the change of target, only simple scale expanding or narrowing will draw into plenty of noises on UAV videos, when aspect ratio of object changes frequently. To deal with this problem, apart from learning a separate 1-dimensional correlation filter to estimate the target scale straightly [30], we propose our adaptive appearance template updating scheme based on CA. Here, the single-layer CA is introduced in our tracker to regularly adjust the aspect ratio of the bounding box. An unlabeled digital image may be then considered as a particular configuration state $S_{I}$ of a cellular automaton, where cellular space I is defined by the array set of image, and initial states $S_{i}$ for ∀i∈I are set to: $l_{i}=0,F_{i}=0,\vec{c_{i}}=RGB_{i}$, where $RGB_{i}$ is the three dimensional vector of pixel’s color in RGB space.

Then, a few number of “object seeds” are selected around the center of target while pixels on the image boundaries are all served as “background seeds” in the current frame. The states of object seeds and background seeds are set to 1 and −1, respectively. It is intuitive to accept that neighbors with more similar color features have a greater influence on the next state of the cell. The similarity of any pair of pixels is measured by a defined distance in RGB color space. So we construct the impact factor matrix $F_{i}={\left[f_{i,j}\right]}_{NxN}$ by defining the impact factor $f_{i,j}$ of pixel i to j as:

(7) $$\begin{array}{lll} f_{i,j}=1-\frac{\left||c_{i},c_{j}|\right|}{\max(c)} & if & j\in NB(i)\\ f_{i,j}=0 & if & j\notin NB(i) \end{array}$$

where $\left||c_{i},c_{j}|\right|$ denotes the Euclidean distance in RGB color space between the pixel i and j, NB(i) is the set of neighbors of cell i. The form of f insures its value within the range [0,1], which is mathematically tractable.

Then, a novel propagation mechanism dependent on CA is proposed to exploit the intrinsic relevance of similar regions through interactions with neighbors:

(8) $$c_{i}(t+1)=\max(c_{j}(t)\cdot F_{ij}(t),c_{i}(t))$$

where $c_{i}(t)$, $c_{i}(t+1)$ denote the value of pixel i in current frame t and the next frame t+1, respectively. What is more, if $c_{i}(t+1)$ is different from $c_{i}(t)$, the value of $l_{i}(t+1)$ will be set the same as $l_{j}(t)$.

To obtain a more precise template, we first identify the center point of the target in the previous frame. A particular patch within its neighborhood is intercepted from the original image manually, which is of a size five times as large as the previous target. Then we repeat the above steps for every pixel in the sampled patch until the states of pixels have no further variation in the current frame. After that, we bag the pixels which states are equal to 1 into a connected domain and take its minimum bounding box. If the area of minimum bounding-box are within the interval [a, b], the connected domain will be considered as the new template to reinitialize our tracker.

**Figure 3:**
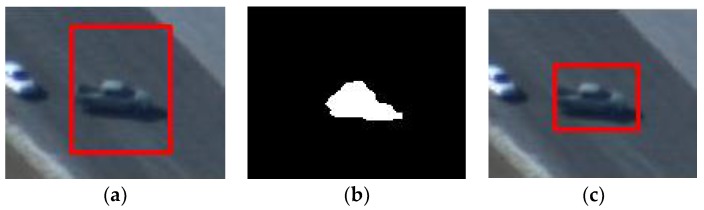
Tracking results of the adaptive appearance template updating scheme. (**a**) Tracking result with the original template; (**b**) Mask of new template obtained by CA; (**c**) Tracking result with the new template.

Therefore, as indicated in Figure 3, the aspect ratio of bounding box can change with the appearance variation adaptively without introducing too much noise, which greatly reduces the risk of model drift. Note that updating with moderate frequency generally leads to an improved tracking result. The appearance of the target usually only changes slightly and the traditional updating strategy in correlation filter is able to handle it well. Under the circumstances, re-initialization densely requires a significant amount of computing resources and may lead to temporal information loss. It is difficult to make a significant improvement over the tracking accuracy merely by the use of excessive dense re-initialization. Of course, when the appearance of the target changes radically, the resulting model draft can cause tracking failure. In this situation, an overly sparse re-initialization cannot introduce new templates in time. From the empirical observations, we choose to reinitialize our correlation filter every 60 frames, trading off between computational efficiency and tracking effectiveness.

### 3.3. Model Updating via High Confidence

No matter the tracking result is accurate or not, the traditional correlation trackers update their models at each frame. In fact, unsupervised updating will lead to model drifting and finally cause a deterministic failure when the occlusion exists severely. To obtain a robust and efficient approximation, we adopt a stability function T [37] to measure the stability of response map R. First, T is defined as:

(9) $$T=\frac{|R_{\max}-R_{\min}{|}^{2}}{mean({\sum_{a,b}\left(R_{a.b}-R_{\min}\right)}^{2})}$$

where $R_{\max}$, $R_{\min}$ and $R_{a,b}$ denote the maximum, minimum and the a−th row b−th column elements of R, respectively.

**Figure 4:**
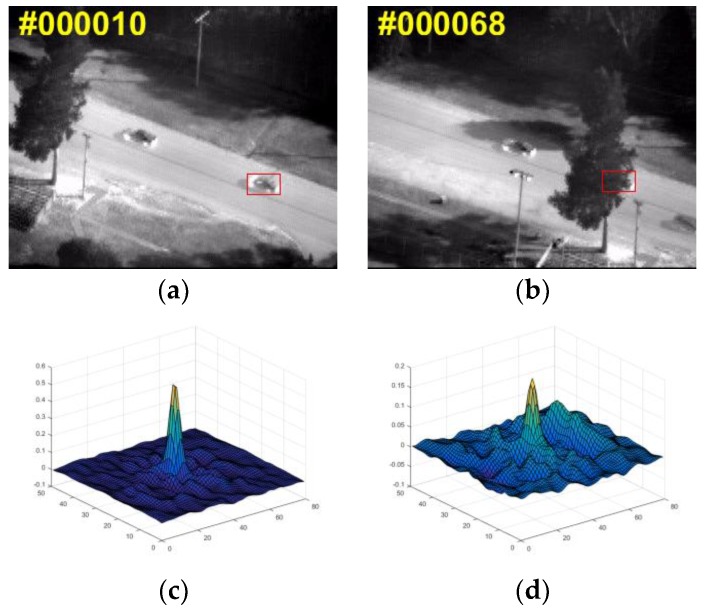
Images and their responses of in different situation. (**a**) Original image without occlusion; (**b**) Original image with serious occlusion; (**c**) Response map of (**a**); (**d**) Response map of (**b**).

Figure 4 shows two original images and their response map in different situation. From Figure 4, it is obvious that the more stable the response map is, the better the location accuracy is. Only when these two criteria $R_{\max}$ and *T* of the current frame are greater than their respective historical average values with certain ratios β1, β2, the tracking result in the current frame is considered to be of high-confidence. Under the circumstances, we will update the numerator $A^{d}$ and the denominator $B^{d}$ of the correlation filter $W^{d}$ in Equation (4) separately, using a moving average:

(10) $$A_{t}^{d}=(1-\eta )A_{t-1}^{d}+\eta Y⊙{\bar{X}}_{t}^{d}$$

(11) $$B_{t}^{d}=(1-\eta )B_{t-1}^{d}+\eta \sum_{i=1}^{D}X_{t}^{i}⊙{\bar{X}}_{t}^{i}$$

(12) $$W_{t}^{d}=\frac{A_{t}^{d}}{B_{t}^{d}+\lambda }$$

where *t* is the frame index and η denotes the learning rate.

When the target is in severely occlusion or totally missing in the current frame, the peak value $R_{\max}$ and stability function *T* may be relative small, our tracker will not update the model in this frame. In this instance, most of interference noise from background are prevented from our model and therefore achieves a robust aerial tracking.

## 4. Experiment and Results

In order to present an objective evaluation about the performance of the proposed approach, the UAV123 dataset [38] is selected to show full results of all chosen trackers. UAV123 provides an evaluation of trackers on more than 100 new fully annotated HD videos captured from a professional grade UAV. This benchmark both complements current benchmarks establishing the aerial component of tracking and provides a more comprehensive sampling of tracking nuisances that are ubiquitous in low-altitude UAV videos. Apart from aspect ratio change (ARC) and fast motion (FM), these video sequences are also affected by several adverse conditions such as background clutter (BC), camera motion (CM), full occlusion (FOC), illumination variation (IV), low resolution (LR), out of view (OV), partial occlusion (POC), similar object (SOB), scale variation (SV), viewpoint change (VC). Thus, the experiments carried out covered all typically challenges typically involved in real-world aerial tracking problems.

The trackers are running on these challenging sequences to test their general ability and also their special scenarios handling. We compare our proposed tracker with nine state-of-art trackers, including ORVT [12], GOTURN [24], BIT [27], DSST [30], fDSST [39], KCFDP [40], SAMF [41], OCT_KCF [42] and CNT [43]. Among these trackers, ORVT is an onboard robust visual algorithm for aerial tracking using a reliable global-local object model.

The proposed tracker is implemented in Matlab2014a on a PC equipped with an Intel i5-7500 processor (four cores, 3.4 GHz clock speed, without hyper-threading technology). In addition, a 16 GB RAM (RAM clock: 2400 MHZ) is utilized without using any sophisticated program optimization. The interval [a, b] is set twice as much as the width and height of the target.

### 4.1. Quantitative Evaluation

We follow the standard evaluation metrics for the tracking algorithms in two aspects: the precision rate and success rate [44]. The precision rate shows the percentage of successfully tracked frames on which the center location error (CLE) of a tracker is within a given threshold (e.g., 20 pixels), and CLE is defined as the average Euclidean distance between the center locations of the targets and the manually labeled ground truths. A tracking result in a frame is considered successful if $\frac{\left|r_{d}\cap r_{t}\right|}{\left|r_{d}\cup r_{t}\right|}>\theta$ for a threshold θ∈(0,1], where $r_{d}$ and $r_{t}$ denote the areas of the bounding boxes of the tracking and the ground truth, respectively, ∩ and ∪ represent the intersection and union of two regions, respectively, and |⋅| denotes the number of pixels in the region. Thus, the success rate is defined as the percentage of frames where the overlap rates are greater than a threshold θ. Normally, the threshold θ is set to 0.5.

**Figure 5:**
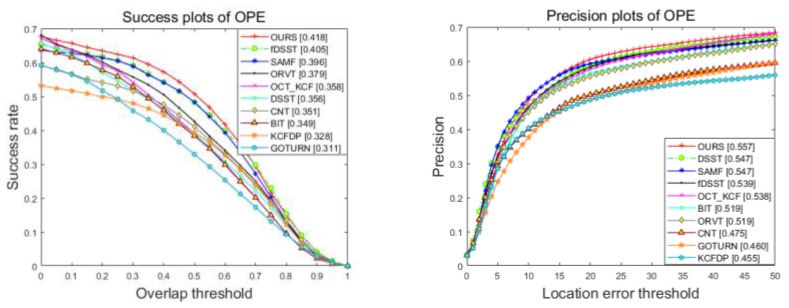
Success (**left**) and precision (**right**) plots of proposed tracker compared with state-of-the-art approaches on UAV123 dataset.

We present the results under one-pass evaluation (OPE) using the average precision and success rate over all sequences. OPE is the most common evaluation method which runs trackers on each sequence for once. It initializes the trackers with the ground truth object state in the first frame and report the average precision or success rate of all the results. Figure 5 shows overall quantitative evaluation on precision and success plots with OPE. Note that our approach provides a gain of 4.0% in success rate as compared to the aerial tracking method ORVT, by achieving a score of 41.8%.

### 4.2. Attribute-Based Comparison

**Figure 6:**
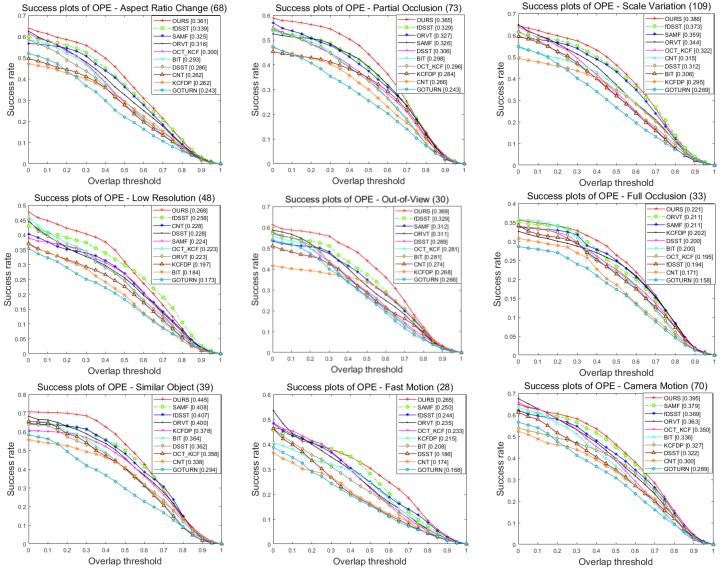

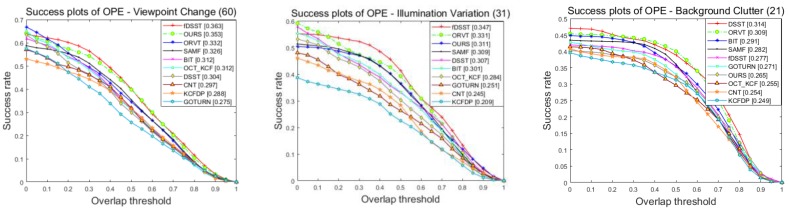
Success plots of our tracker compared with state-of-the-art approaches on UAV123 dataset.

**Figure 7:**
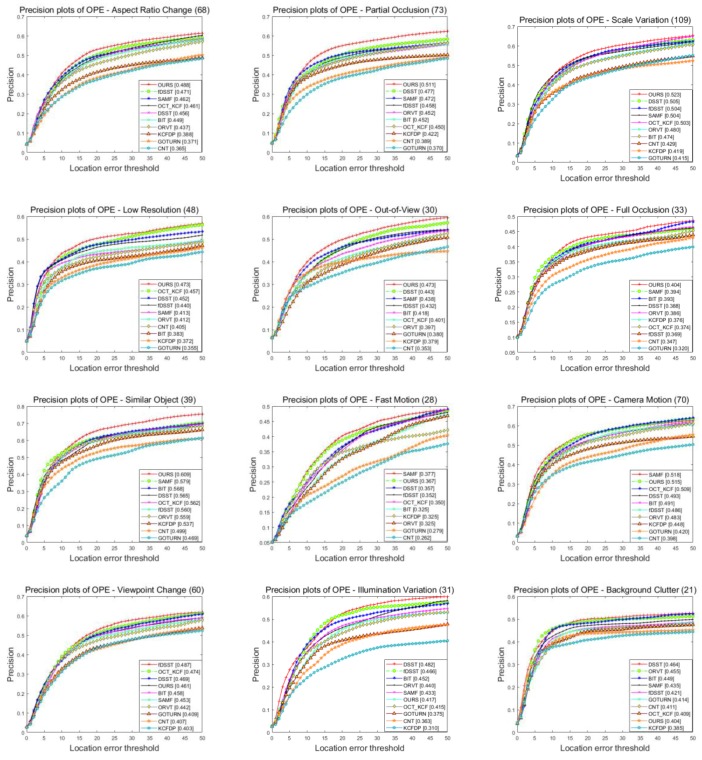
Precision plots of our tracker compared with state-of-the-art approaches on UAV123 dataset.

We also perform an attribute-based comparison with other methods on the UAV123 dataset. Figure 6 and Figure 7 show the success plots and precision plots of twelve respective attributes on the precision and success rates, respectively. The overall results with different attributes are summarized in Table 1 and Table 2, which show the averaged rates of the success plots and those of the precision plots, respectively. As can be seen from these results, our tracker always performs reliably and can achieve optimal or at least close to optimal solution in most cases. Specifically, for the amplified challenging factors in aerial tracking, including CM, LR, SV, ARC, FM, FOC and POC, our tracker achieves satisfactory results, benefitting from the robustness of fused features as well as the efficiency of appearance template and model updating strategy. For videos with fast moving objects, camera motion and low resolution, the fused features have more strong abilities to capture the information from object and therefor gain better results compared with the classic single-feature trackers. In addition, when aspect ratio of object changes significantly, our adaptive appearance template updating strategy can adjust the template to the appearance of object. Moreover, the high confidence model updating method prevents the noise from background as much as possible when serious occlusion exists in aerial video. However, our tracker may not perform well when dealing with background clutter and illumination variation. It is probably because these challenges have created serious problems for CA, which result in a partial or inexact template and finally lead to mistaken tracking.

### 4.3. Qualitative Evaluation

**Figure 8:**
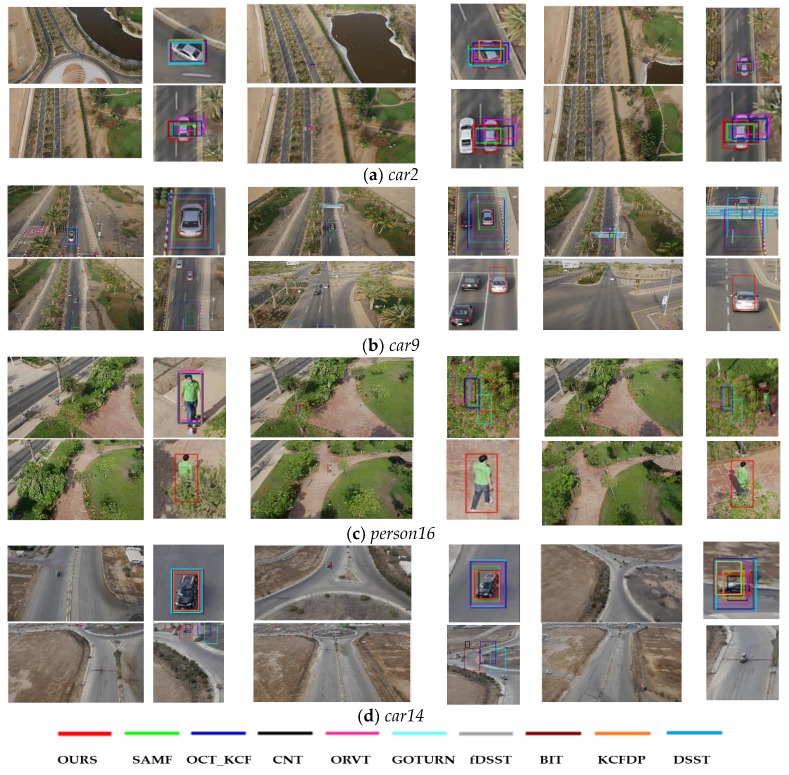
Tracking results of different methods on four representative sequences.

For qualitative evaluation, we select four representative sequences from UAV123 dataset, on which we compared our tracker against five state-of-the-art methods to validate the ability of the proposed approach. Sequences are shown in Figure 8 (from top to down are sequences *car2*, *car9*, *car14*, *person16*, respectively). Because the targets in the dataset are rather small, we present the tracking results and their partial enlargements to show the comparison of the tracking results of each algorithm more clearly.

As shown in Figure 8a, the aspect ratio of the target has changed significantly, due to car movement. Only our tracker can adaptively adjust to this variation, while other algorithms still try to track the target with the original aspect ratio. As our tracker introduces less noise from the background, model drifting risk is reduced. Regarding the sequence *car9*, similar objects and scale validation exist over a long period. Apart from that, the target has been severely occluded by a road sign. In such a situation, only our method can track the target stably and outperform on coverage, benefitting from the high confidence model updating strategy. The tracking results on the sequence *person16* are shown in Figure 8c. In this sequence, the target suffers from partial or full occlusion and has quite similar color with the background. Under such interference no other method can track the target expect for ours. Owing to the employed stability function, our method is able to prevent low confidence model updating and track the target even after such complex background disturbances. Figure 8d illustrates that only our tracker and the KCFDP tracker can adapt well to the aspect ratio and target scale changes on the sequence with low resolution. Our tracker achieves better scale and position accuracy in comparison with the KCFDP algorithm. However, after violently shaking of the camera, the target is out of view for a long time. At this moment, all the trackers in the experiment drift away.

Qualitative analysis shows that our method can effectively address the problems in aerial tracking, especially the SV, OCC and ARP. These robust results are attributed to the model updating via high confidence as well as the adaptive appearance template updating scheme. Moreover, the fused features also make a great contribution to improve the tracking results on the UAV videos with low resolution.

## 5. Discussion

### 5.1. Speed Performance

For practical applications of aerial tracking, the computational efficiency of trackers also needs to be taken into account. Table 3 lists the running speed of each tracker on nine sequences of the UAV123 dataset, and the average speeds over all of the sequences are shown in the last row.

As we can see, the fDSST tracker achieves the highest running speed which is almost 99 fps and the biologically inspired BIT tracker performs well in terms of running efficiency, too. However, CNN-based CNT and GOTURN trackers show low running efficiencies on all of the nine test sequences, which may not meet the standard of real-time running. It is also worthwhile to note that our tracker can meet the real-time requirements, while gaining the outstanding results on both success rate and precision rate. This owes much to the robustness of fused feature and the efficiency of adaptive appearance template updating strategy. Under this basis, we are trying to find an optimization method to speed up our tracker. Meanwhile, our code will be run on a more appropriate running platform to test its portability, preparing for the real-world application.

### 5.2. Effect of Adaptive Appearance Template Updating

**Figure 9:**
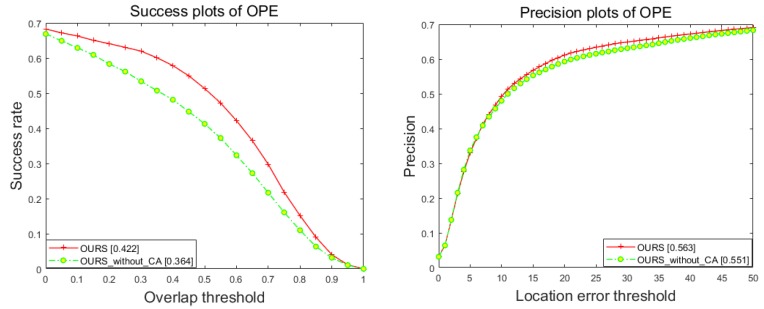
The success and precision plots of our tracker compared with which without adaptive appearance template updating strategy on the UAV123 dataset.

As mentioned in Section 3.2, when the aspect ratio of object is changing, classic correlation trackers are only able to simply expand or narrow the scale, which will draw into plenty of noises from background in aerial tracking. To deal with this problem, we employ an adaptive appearance template updating strategy in our trackers. We analyze the impact of introducing CA to dig out the interrelationship between pixels of object and adjust the template to the appearance change of target on the UAV videos. Figure 9 shows the tracking performance in success plots and precision plots. From this figure we can see that the performance of our tracker improves considerably as compared to the corresponding version of it without employing the adaptive appearance updating strategy. Our results suggest that noises can be significantly reduced with adaptive appearance template updating strategy, while preserving the satisfying tracking performance.

## 6. Conclusions

In this paper, we propose a novel method to achieve robust aerial tracking. Our approach is based on learning separate discriminative correlation filters for translation and scale estimation. Furthermore, the fused features consisting of CN and HOG features are utilized to improve the ability of our tracker to capture a wealth of information. In addition, we design a series of strategies to adaptively update the appearance template of our tracker based on CA. This allows the template to adapt the aspect ratio change of target and bring in less disturb from background. Notably, a stability function is introduced to update the model in a more reliable way. Finally, we conduct extensive experiments on the UAV123 dataset. The results clearly demonstrate that our approach achieves the state-of-the-art tracking accuracy. Future work includes investigating more powerful fused features to combine intensity and color information. Another research direction is to exploit efficient deep neural network models (e.g., the light-weight neural network), in an effort to achieve more robust aerial tracking for real time applications.

## Figures and Tables

**Table 1:** Overall precision rates on different attributes, where the entries in red denote the best results and the ones in green indicate the second best.

	Ours	BIT	fDSST	KCFDP	SAMF	DSST	ORVT	CNT	GOTURN	OCT_KCF
SV	**0.523**	0.474	0.504	0.419	0.504	**0.505**	0.480	0.429	0.415	0.503
ARC	**0.488**	0.449	**0.471**	0.388	0.462	0.456	0.437	0.365	0.371	0.461
LR	**0.473**	0.383	0.440	0.372	0.413	0.452	0.412	0.405	0.355	**0.457**
FM	**0.367**	0.325	0.352	0.325	**0.377**	0.357	0.325	0.262	0.279	0.350
FOC	**0.404**	0.393	0.369	0.376	**0.394**	0.388	0.386	0.347	0.320	0.374
POC	**0.511**	0.452	0.458	0.422	0.472	**0.477**	0.452	0.386	0.370	0.450
OV	**0.473**	0.418	0.432	0.379	0.438	**0.443**	0.397	0.353	0.380	0.401
BC	0.404	0.449	0.421	0.385	0.435	**0.464**	**0.455**	0.411	0.414	0.409
IV	0.417	0.452	**0.466**	0.310	0.433	**0.482**	0.440	0.363	0.375	0.415
VC	0.461	0.458	**0.487**	0.403	0.453	0.469	0.442	0.407	0.409	**0.474**
CM	**0.515**	0.491	0.486	0.448	**0.518**	0.493	0.483	0.389	0.420	0.509
SOB	**0.609**	0.568	0.560	0.537	**0.579**	0.565	0.559	0.499	0.469	0.562
Overall	**0.557**	0.519	0.539	0.455	**0.547**	**0.547**	0.519	0.475	0.460	0.538

**Table 2:** Overall success rates on different attributes, where the entries in red denote the best results and the ones in green indicate the second best.

	Ours	BIT	fDSST	KCFDP	SAMF	DSST	ORVT	CNT	GOTURN	OCT_KCF
SV	**0.386**	0.306	**0.373**	0.295	0.359	0.312	0.344	0.315	0.269	0.322
ARC	**0.361**	0.293	**0.339**	0.262	0.325	0.286	0.316	0.262	0.243	0.300
LR	**0.268**	0.184	**0.258**	0.197	0.224	0.228	0.223	0.228	0.173	0.223
FM	**0.265**	0.208	0.244	0.215	**0.250**	0.186	0.235	0.174	0.168	0.223
FOC	**0.221**	0.200	0.194	0.202	**0.211**	0.200	**0.211**	0.171	0.158	0.195
POC	**0.365**	0.298	**0.329**	0.284	0.326	0.306	0.327	0.266	0.243	0.296
OV	**0.369**	0.281	**0.329**	0.268	0.312	0.289	0.311	0.274	0.266	0.281
BC	0.265	0.291	0.277	0.249	0.282	**0.314**	**0.309**	0.254	0.271	0.255
IV	0.311	0.301	**0.347**	0.209	0.309	0.307	**0.331**	0.245	0.251	0.284
VC	**0.353**	0.312	**0.363**	0.288	0.326	0.304	0.332	0.297	0.275	0.312
CM	**0.395**	0.336	0.369	0.327	**0.379**	0.332	0.363	0.300	0.289	0.350
SOB	**0.445**	0.364	0.407	0.378	**0.408**	0.362	0.400	0.338	0.294	0.358
Overall	**0.418**	0.349	**0.405**	0.328	0.396	0.356	0.379	0.351	0.311	0.358

**Table 3:** Running speed (frame per second) of each tracker on sequences from UAV123 dataset, where the entries in red denote the best results and the ones in green indicate the second best.

	Target Size	Ours	BIT	fDSST	KCFDP	SAMF	DSST	ORVT	CNT	GOTURN	OCT_KCF
boat6	27 × 16	33	**121**	134	30	6	**124**	32	0.91	6.10	116
car1	69 × 89	24	**94**	62	43	7	**92**	23	0.75	8.74	16
car2	39 × 21	31	105	**165**	17	6	105	26	1.00	0.36	**172**
car9	99 × 169	7	12	31	18	10	9	**43**	0.73	0.42	**64**
car14	43 × 68	14	**54**	**89**	36	5	38	17	0.76	0.65	21
person2	50 × 111	10	**42**	**72**	15	5	19	29	0.82	1.39	24
person6	33 × 95	11	**49**	**77**	22	6	33	27	1.38	0.71	20
person16	33 × 71	15	**58**	**101**	29	5	45	14	0.75	12	40
person22	17 × 47	24	97	**158**	46	6	92	28	0.73	9.46	**196**
